# Rare taxa mediate microbial carbon and nutrient limitation in the rhizosphere and bulk soil under sugarcane–peanut intercropping systems

**DOI:** 10.3389/fmicb.2024.1403338

**Published:** 2024-05-30

**Authors:** Yue Fu, Xiumei Tang, Tingting Sun, Litao Lin, Lixue Wu, Tian Zhang, Yifei Gong, Yuting Li, Haining Wu, Jun Xiong, Ronghua Tang

**Affiliations:** ^1^College of Agronomy, Guangxi University, Nanning, Guangxi, China; ^2^Key Laboratory of Agro-Environment and Agro-Product Safety, Guangxi University, Nanning, China; ^3^Guangxi Academy of Agricultural Sciences, Cash Crops Research Institute, Nanning, Guangxi, China; ^4^Center for Ecological Civilization Research, Chinese Research Academy of Environmental Sciences, Beijing, China

**Keywords:** microbial nutrient limitation, intercropping, rhizosphere, microbial community, rare taxa

## Abstract

**Introduction:**

Microbial carbon (C) and nutrient limitation exert key influences on soil organic carbon (SOC) and nutrient cycling through enzyme production for C and nutrient acquisition. However, the intercropping effects on microbial C and nutrient limitation and its driving factors between rhizosphere and bulk soil are unclear.

**Methods:**

Therefore, we conducted a field experiment that covered sugarcane–peanut intercropping with sole sugarcane and peanut as controls and to explore microbial C and nutrient limitation based on the vector analysis of enzyme stoichiometry; in addition, microbial diversity was investigated in the rhizosphere and bulk soil. High throughput sequencing was used to analyze soil bacterial and fungal diversity through the 16S rRNA gene and internal transcribed spacer (ITS) gene at a phylum level.

**Results:**

Our results showed that sugarcane–peanut intercropping alleviated microbial C limitation in all soils, whereas enhanced microbial phosphorus (P) limitation solely in bulk soil. Microbial P limitation was also stronger in the rhizosphere than in bulk soil. These results revealed that sugarcane-peanut intercropping and rhizosphere promoted soil P decomposition and facilitated soil nutrient cycles. The Pearson correlation results showed that microbial C limitation was primarily correlated with fungal diversity and fungal rare taxa (*Rozellomycota, Chyltridiomycota*, and *Calcarisporiellomycota*) in rhizosphere soil and was correlated with bacterial diversity and most rare taxa in bulk soil. Microbial P limitation was solely related to rare taxa (*Patescibacteria* and *Glomeromycota*) in rhizosphere soil and related to microbial diversity and most rare taxa in bulk soil. The variation partitioning analysis further indicated that microbial C and P limitation was explained by rare taxa (7%–35%) and the interactions of rare and abundant taxa (65%–93%).

**Conclusion:**

This study indicated the different intercropping effects on microbial C and nutrient limitation in the rhizosphere and bulk soil and emphasized the importance of microbial diversity, particularly rare taxa.

## 1 Introduction

Microorganisms are the main biota groups in soil and play a crucial role in agroecosystems by delivering essential functions such as storage of soil organic carbon (SOC) and nutrient cycling (Wieder, 2014). They produce diverse enzymes for the breakdown of soil organic matter (SOM) and the mineralization of soil nutrients, such as nitrogen (N) and phosphorus (P), to maintain plant and microbial growth (Sinsabaugh et al., 2008, 2009). The enzyme production is ascribed to soil nutrient availability and microbial biomass demands, with high enzyme activity in resource-poor environments (Gomez et al., 2020; Zhou L. H. et al., 2020). The C-, N-, and P-acquiring enzyme activity and their stoichiometry thus reflect the magnitudes of microbial C and nutrient limitation (N vs. P) (Qiu et al., 2021; Zheng et al., 2022). For example, higher C:N:P-requiring enzymes show stronger microbial C limitation relative to nutrients, which regulates SOM decomposition and soil nutrient cycling (Zheng et al., 2022). Thus, the response of microbial C and nutrient limitation to different agricultural management practices is of great importance in understanding agroecosystem nutrient cycling and supporting the development of sustainable agricultural management practices.

Intercropping is a sustainable agricultural practice to promote belowground productivity, yield, and ecosystem services, such as soil C, N, and P cycles (Cong et al., 2015; Li et al., 2020). This is due to the functional compensability of multiple crops to increase the land equivalent ratio with high water, air, and sunlight, as well as nutrient use efficiency by increasing the soil enzyme activities in the same land compared to sole crops (Li et al., 2020; Curtright and Tiemann, 2021). Compared with sole crop, on the one hand, the increase in soil nutrient and root-derived C (root exudates) induced by intercropping might alleviate microbial C and nutrient limitation by increasing fertilizer use efficiency (Steinauer et al., 2015; Tang X. Y. et al., 2021). On the other hand, intercropping-induced frequent interactions in root–soil–microbes systems and fast-growing microbes appear to enhance nutrient competition between microbes–microbes and microbes–crops, which is likely to regulate microbial C and nutrient limitation (Hilbig and Allen, 2015; Kuster et al., 2016; Feng et al., 2021). It is therefore essential to explore the intercropping effects on microbial C and nutrient limitation, which could further affect soil nutrient uptake and plant biomass productivity and yields (Tang X. Y. et al., 2021). Although many studies observed the intercropping effects on enzyme activity, most of them considered the bulk soil and few studies focused on the rhizosphere, particularly, the differences in intercropping effects on microbial C and nutrient limitation between rhizosphere and bulk soil (Curtright and Tiemann, 2021; Shi et al., 2022; dos Santos Bastos et al., 2023).

The rhizosphere, as the interface of soil–microbes–plant, directly controls nutrient and C decomposition and reutilization, thereby regulating plant productivity. Given the root disturbance and selection, the rhizosphere, as a microbial hotspot, has higher microbial activity and diversity than those in bulk soil (Elmajdoub et al., 2014; Pang et al., 2021; Chen et al., 2023). Additionally, the changes in enzyme activities between rhizosphere and bulk soil are dependent on the differences in nutrient and labile C in these two soils (Ren et al., 2021; Chen et al., 2023). For example, the larger intercropping effect on increasing enzyme activity is visible in nutrient-poor soils, such as bulk soil (Curtright and Tiemann, 2021). This implies that microbial C and nutrient limitation in the rhizosphere and bulk soil would have divergent responses to intercropping through changes in nutrient and labile C. Therefore, assessing the intercropping effects on microbial C and nutrient limitation in both rhizosphere and bulk soil would improve the understanding of soil nutrient and C cycling in root-soil systems in response to intercropping, which offers benefits for the development of sustainable agricultural management practices.

Microbial C and nutrient limitation are highly controlled by the microbial community (Creamer et al., 2015; Yao et al., 2018), such as rare and abundant taxa, which are different between rhizosphere and bulk soil (Wang et al., 2022; Zhang G. Z. et al., 2022). Rare taxa, groups with a narrower niche breadth, are more sensitive to environmental disturbances and might be present at specific locations or be exclusive to arable farming practices (Banerjee et al., 2024). Many studies show that compared with abundant taxa, rare taxa (e.g., *Actinobacteria* and *Cyanobacteria*) play a more important role in C and nutrient cycles, especially in rhizosphere owing to the root-induced selection and disturbance effects (Wei et al., 2019; Zhang et al., 2021; Wang et al., 2022). Furthermore, soil property, such as nutrient availability, is another vital factor controlling microbial resource limitation (Chen et al., 2019; Cui et al., 2021). Microbes are more C- or nutrient-limited in resource-poor environments with higher soil C:N ratio (Cui et al., 2021). This is because microbes, especially for rare taxa, prefer fertile soils with low soil C:N ratio substrates, which are easily decomposed and utilized (Liu et al., 2023). However, the intercropping effects on rare and abundant taxa, as well as the relative contributions of the microbial community (rare and abundant taxa) and soil properties to microbial C and nutrient limitation in the rhizosphere and bulk soil, are limited. This limitation would shed insights into the contributions of biotic and abiotic factors to microbial nutrient limitation in root–soil systems under intercropping practices.

Sugarcane–peanut intercropping is widely introduced to increase crop yield and soil fertility (Tang et al., 2021a, 2022). In this study, we aimed to compare the intercropping effects on microbial C and nutrient limitation and the relative contributions of rare and abundant taxa in the rhizosphere and bulk soil. We hypothesize that (1) sugarcane/peanut intercropping might decrease microbial C and nutrient limitation due to more C and nutrient input via legume biological N_2_-fixing than monoculture; (2) the intercropping effects on microbial C and nutrient limitation would be lower in rhizosphere soil than in bulk soil with poor resource; and (3) microbial C and nutrient limitation might be mostly regulated by rare taxa in rhizosphere soil and abundant taxa in bulk soil. This is because compared to abundant taxa, rare taxa depend on nutrient availability to a larger extent (Xu et al., 2021a; Lin et al., 2022), and thus rare taxa might primarily regulate microbial C and nutrient limitation in rhizosphere with richer root-derived substrates input.

## 2 Materials and methods

### 2.1 Site description

This field experiment was conducted from 2021 to 2023 at the Lijian Scientific Base of the Guangxi Academy of Agricultural Sciences (E108°3′40″, N23°14′58″), Nanning City, Guangxi Zhuang Autonomous Region (GZAR), China. This study site is located in the southwest of China with a subtropical climate zone. The mean annual temperature and precipitation were 22°C yr^−1^ and 1322 mm yr^−1^, respectively, obtained from the World Weather Information Service (https://worldw-eather.wmo.int/en/home.html) based on the longitude and latitude of the studied location. The soil type was classified as red soil with a loam texture. Before the field experiment, soil water content (SWC) was 14.39%, pH (H_2_O) was 6.75, and SOC, total N (TN), and P (TP) were 18.52, 1.29, and 1.26 g kg^−1^ in 0–20 cm soil layer, respectively. The provided sugarcane and peanut varieties were, respectively, “Guitang44” and “Guihua376” from the Cash Crops Research Institute of the Guangxi Academy of Agricultural Sciences (Tang et al., 2022).

### 2.2 Experiment design

**Figure 1:**
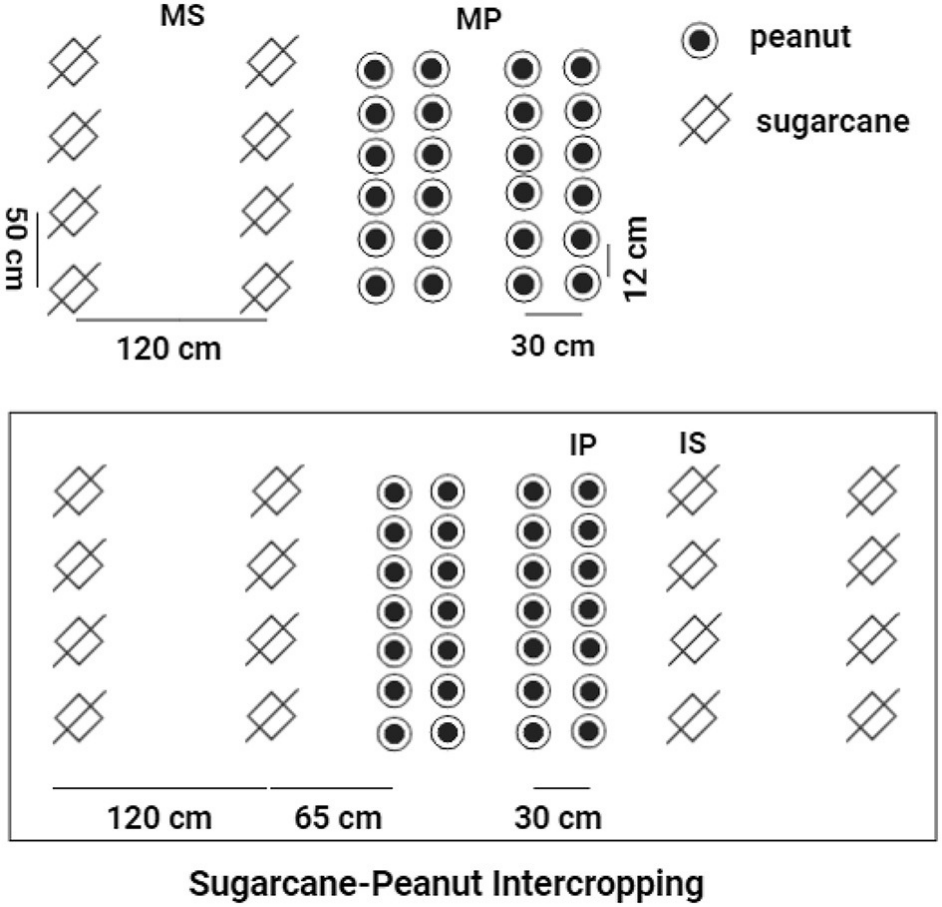
The schematic diagram of sugarcane–peanut intercropping. MS, monoculture sugarcane soil; IS, sugarcane soil peanut soil in sugarcane–peanut system; MP, monoculture peanut soil; IP, peanut soil in sugarcane–peanut system.

This study included four treatments of cropping systems, including (1) monoculture peanut (MP); (2) monoculture sugarcane (MS); (3) sugarcane soil in sugarcane–peanut intercropping systems (IS); and (4) peanut soil in sugarcane–peanut intercropping systems (IP). For MS, sugarcane was planted with a row spacing of 1.2 m and a plant width of 50 cm. For MP, the row spacing of peanut was 30 cm with a plant spacing of 12 cm. For IS and IP, the line spacing between sugarcane and peanut was 65 cm (sugarcane: peanut = 2:4) in intercropping systems. The line spacing for sugarcanes was 1.2 m and that for the intercropped peanuts was 30 cm (Figure 1). The experiment was conducted in plots (10 m × 11.7 m) in a randomized design with six replicates in each treatment. All peanut treatments received 450 kg ha^−1^ special compound fertilizer (N-P_2_O_5_-K_2_O = 15-15-15) and 750 kg ha^−1^ fused calcium–magnesium phosphate fertilizer (P_2_O_5_ = 18%). All sugarcane treatments were fertilized with 2,250 kg ha^−1^ special compound fertilizer (N-P_2_O_5_-K_2_O = 15-15-15). The input rates of N–P_2_O_5_-K_2_O fertilizers were widely recommended to promote the rapid growth of sugarcane and peanut cultivation, which was described in detail in the previous studies (Pang et al., 2021; Tang et al., 2021b, 2022).

### 2.3 Soil sampling

Soils were sampled after the peanut harvest on 11 July 2023. We collected two soils: rhizosphere and bulk soil (0–20 cm soil layer) from peanut and sugarcane from the above four cropping systems. The bulk soil was collected far away from 20 cm of crops. Rhizosphere soil that lightly adhered to plant roots was obtained with a brush (Jiang et al., 2017). In total, 48 soil samples were finally collected (four treatments × two soils × six replicates). All soil samples were delivered to the laboratory immediately and were sieved into 2 mm with the removal of visible stones and root residues. In total, 20 g of soil were stored at −20°C for microbial analysis, and the rest of the soil was stored at 4°C for soil physicochemical analysis.

### 2.4 Physicochemical properties of the soil

The water content of the soil was determined by oven-drying. Soil pH was extracted by a 1:5 fresh soil:water ratio (mass:volume) and was measured using a pH meter. SOC was determined by the potassium dichromate oxidation method. Soil TN was measured by the Kjeldahl method (Bremner and Mulvaney, 1982). Soil total P (TP) was extracted by H_2_SO_4_-HClO_4_ and then was measured by the molybdenum blue method at 700 nm emission (Page, 1982) using an ultraviolet spectrophotometer (UV2600, Shimadzu, Japan).

### 2.5 Soil microbial biomass and community composition

Soil microbial biomass C (MBC), N (MBN), and P (MBP) were analyzed with fresh soil samples using the chloroform fumigation–extraction method (Vance et al., 1987).

Soil total DNA was extracted from 1 g of frozen soil by the E.Z.N.A.^®^ soil DNA Kit (Omega Bio-tek, Norcross, GA). A NanoDrop 2000 spectrophotometer (Thermo Scientific, Waltham, MA) and 2% agarose gel electrophoresis were used to assess the quantity and quality of DNA. The primer pairs 515F/907R (5′-GTGCCAGCMGCCGCGG-3′/5′-CCGTCAATTCMTTTRAGTTT-3′) (Xu et al., 2021b) and ITS 1F/ITS 2R (5′-CTTGGTCATTTAGAGGAAGTAA-3′/5′-GCTGCGTTCTTCATCGATGC-3′) (Zhou et al., 2023) were used to amplify the bacterial 16S rRNA gene and fungal ITS gene, respectively. Polymerase chain reaction (PCR) was performed in a thermal cycler (ABI GeneAmp 9700) with a volume of 20 μl containing 4 μl of FastPfu Buffer (5 × ), 0.2 μl of BSA, 0.8 μl of forward primer (5 μM), 0.8 μl of reverse primer (5 μM), 2 μl of dNTPs (2.5 mM), 10 ng of DNA template, 0.4 μl of FastPfu Polymerase, and 11.8 μl of ddH_2_O. The reaction conditions were as follows: 3 min of initial denaturation at 95°C, 35 cycles of 30 s at 95°C, 30 s at 55°C, and 45 s at 72°C, and a final elongation for 10 min at 72°C.

Purified amplicons were pooled in equimolar amounts and paired-end sequenced on an Illumina PE250 platform (Illumina, San Diego, CA) according to the standard protocols by Majorbio Bio-Pharm Technology Co. Ltd. (Shanghai, China). DADA2 denoising was used to remove low-quality reads (Callahan et al., 2016), and then the eligible merged sequences were clustered into amplicon sequence variants (ASVs). Taxonomic assignment of ASVs was performed using the basic local alignment tool (BLAST) consensus taxonomy classifier implemented in Qiime2 and the SILVA 16S rRNA database (v138). To minimize the effects of sequencing depth on alpha and beta diversity measure, the number of sequences from each sample was rarefied to 96,908 sequences and 6,738 sequences per sample randomly selected from bacterial 16S rRNA and fungal ITS datasets, respectively, which still yielded an average Good's coverage of 99.0%. Shannon indices, as microbial alpha diversity, were calculated according to the 97% amplicon sequence variant (ASV) similarity of the sequences. The raw sequencing reads were deposited into the NCBI Sequence Read Archive (SRA) database under accession number: PRJNA1051854.

### 2.6 Soil enzyme activity

The frequently determined C, N, and P acquisition enzymes, including β-1,4-glucosidase (BG, C-acquiring enzyme), β-1,4-Nacetylglucosaminidase (NAG, N-acquiring enzyme), leucine aminopeptidase (LAP, N-acquiring enzyme), and acid phosphatase (AP, P-acquiring), were considered in this study (Sinsabaugh et al., 2008, 2009). The potential activities of BG, NAG, LAP, and AP were determined following the protocol of our previous study (Sun et al., 2023). First, a 1.50 g of fresh soil sample was suspended in 150 ml of sodium acetate buffer (30 mmol L^−1^, pH = 5.3) and completely homogenized in a blender for 2 min. The homogenized soil slurries (200 μl) were next added to a black 96-well microplate with eight replicates for each sample. Then, 50 μl of 200 μmol L^−1^ substrates labeled by 4-methylumbelliferone (MUB) for BG, NAG, and AP, or 7-amino-4-methylcoumarin (AMC) for LAP were added to each well and mixed with soil slurries slightly. The standard curve was conducted for each soil slurry sample with a concentration gradient of MUB or AMC (0, 0.1, 0.2, 0.5, 1, 2, 2.5, and 5 mmol L^−1^). All microplates were incubated at 25°C in the dark for 3 h. Finally, the intensity of fluorescence was determined at 365 nm excitation and 460 nm emission by using a microplate reader (Infinite M2000, Tecan, Männedorf, Switzerland). Enzyme activities were calculated and expressed as nmol g^−1^ dry soil h^−1^.

### 2.7 Statistical analysis

The soil microbial resource limitation was calculated by the enzymatic vector analysis (Moorhead et al., 2016) according to Equations 1 and 2 as follows:


(1) $$\begin{array}{l} \text{Vector length}=\text{SQRT}\left({\text{x}}^{2}+{\text{y}}^{2}\right) \end{array}$$

(2) $$\begin{array}{l} \text{Vector angle}=\text{Degrees }\left(\text{Atan2}\left(\text{x},\text{y}\right)\right) \end{array}$$

*x* indicates the proportional activity of C:P acquiring enzymes [ln(BG): ln(BG + AP)], and *y* indicates the proportional activity of C:N acquiring enzymes [ln(BG): ln(BG + NAG + LAP)]. The vector length represents microbial C limitation, and the vector angle indicates microbial P vs. N limitation (Moorhead et al., 2013, 2016). This approach has been widely used to explore microbial resource limitation in response to climate changes and agricultural management (Jing et al., 2020; Zhang S. H. et al., 2022; Sun et al., 2023). Microorganisms are more C-limited with higher vector length, whereas they are relatively P-limited when vector angle >45°, and conversely, N-limited.

Microbial alpha diversity was represented by Shannon indices, which were common indicators of microbial species richness and diversity and were widely used in previous studies (Větrovský et al., 2019; Xu et al., 2021b). Microbial beta diversity was conducted by the non-metric multidimensional scaling (NMDS) analysis based on the Bray–Curtis dissimilarity matrix using the “vegan” package and the first component (NMDS1) was used for the latter analysis (Bahram et al., 2018; Domeignoz-Horta et al., 2020). An analysis of similarities (ANOSIM) was used to evaluate the significant differences in soils (rhizosphere and bulk soil) and cropping systems (monoculture and intercropping) presented in NMDS (Ju et al., 2023; Zhou et al., 2023). The abundant and rare taxa were grouped by the relative abundance approach with the threshold of 1% at the phylum level, which was widely discussed in previous studies (Xu et al., 2021a; Zhang G. Z. et al., 2022; Liu et al., 2023; Wang et al., 2024). The comparisons of the intercropping effects on soil properties, microbial alpha diversity, microbial C, and nutrient limitation between rhizosphere and bulk soil were conducted by one-way ANOVA. The multiple-way ANOVA test was further approached to explore the interactions between crop types (sugarcane vs. peanut), soils (rhizosphere vs. bulk soil), and cropping systems (intercropping vs. monoculture) on soil and microbial properties. Pearson's correlation was used to explore the relationships of biotic (soil properties) and abiotic factors (microbial biomass, microbial community composition, and rare and abundant taxa) with microbial C and nutrient limitation. Furthermore, the variation partitioning analysis (VPA) was conducted to assess the relative contributions of rare and abundant taxa on microbial C and nutrient limitation by using multiple linear regression models due to the weak impact of soil properties (Jing et al., 2020; Sun et al., 2023). All analyses were conducted with R (version 4.2.0) by using the “ggpmisc” package for linear regression models and “modEvA” packages for VPA analyses (Jing et al., 2020; Sun et al., 2023).

## 3 Results

### 3.1 Intercropping effects on soil properties in the rhizosphere and bulk soil

Compared with monoculture systems, sugarcane–peanut intercropping decreased SWC content and soil pH in all soils, especially in the rhizosphere soil with a reduction of 2 units of pH (Supplementary Table S1). Soil pH was also reduced by 0.8 units in the rhizosphere than that in the bulk soil (Supplementary Table S1). Intercropping increased SOC by 16.18%−22.73% relative to monoculture treatment in sugarcane rhizosphere and bulk soil. Furthermore, intercropping increased TN and TP, especially in peanut soils, with an increase of 112.69%−115.27% of TN and 10.37%−33.88% of TP in the rhizosphere and bulk soil. Intercropping did not change soil C:N, C:P, and N:P ratios in all soils, except for MP and IP treatments in bulk soil.

### 3.2 Intercropping effects on microbial community composition and biomass in the rhizosphere and bulk soil

**Figure 2:**
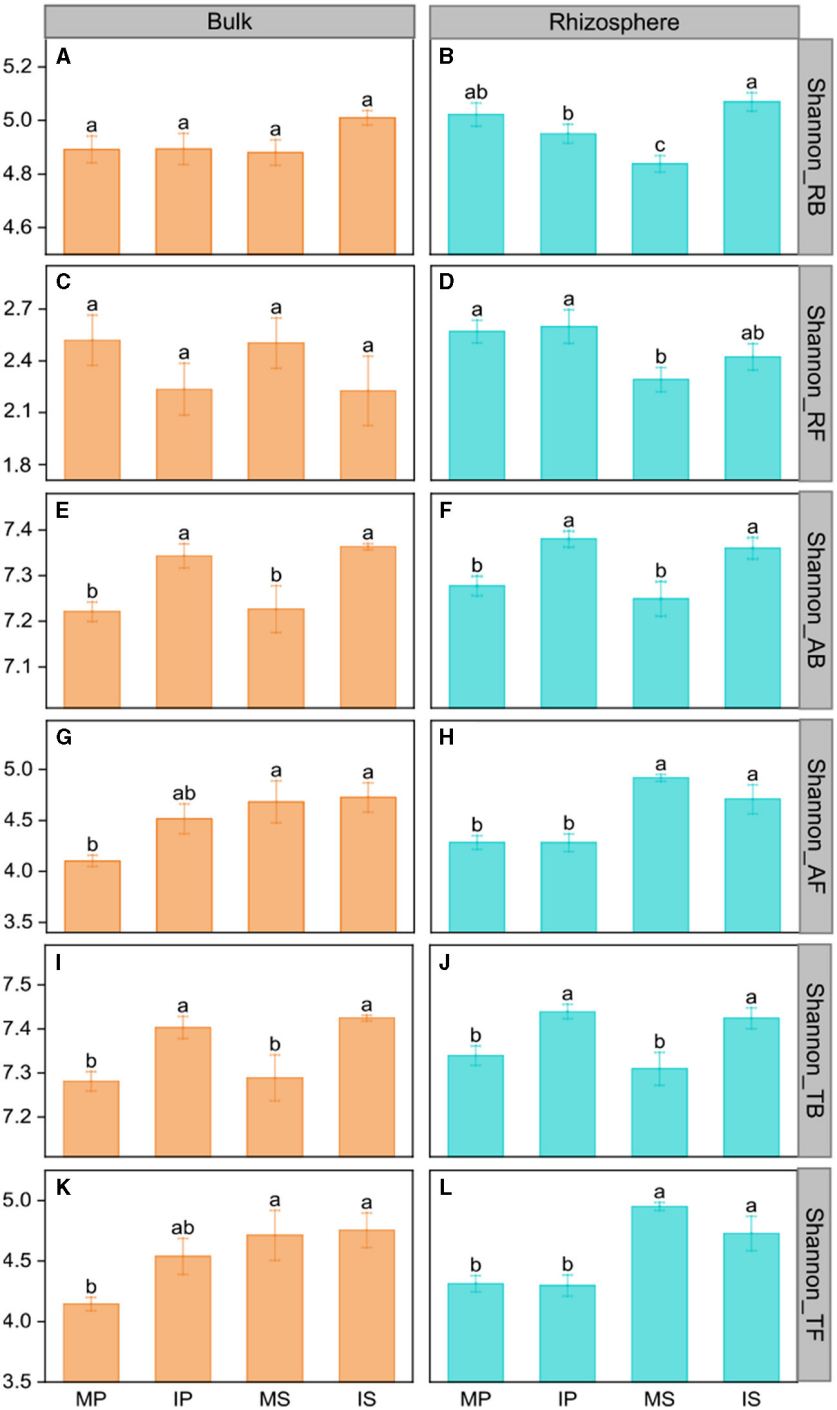
The intercropping effects on Shannon diversity of the microbial community in the rhizosphere **(B**, **D**, **F**, **H**, **J**, **L)** and bulk soil **(A**, **C**, **E**, **G**, **I**, **K)** at the phylum level. MS, monoculture sugarcane; IS, intercropping sugarcane; MP, monoculture peanut; IP, intercropping peanut. Shannon_RB, Shannon_RF, Shannon_AB, Shannon_AF, Shannon_TB, and Shannon_TF indicate the Shannon diversity of rare bacteria and fungi, abundant bacteria and fungi, and total bacteria and fungi, respectively. The lowercase letters indicate significant differences among different cropping patterns in both rhizosphere and bulk soil by LSD test at *P* < 0.05, respectively.

**Figure 3:**
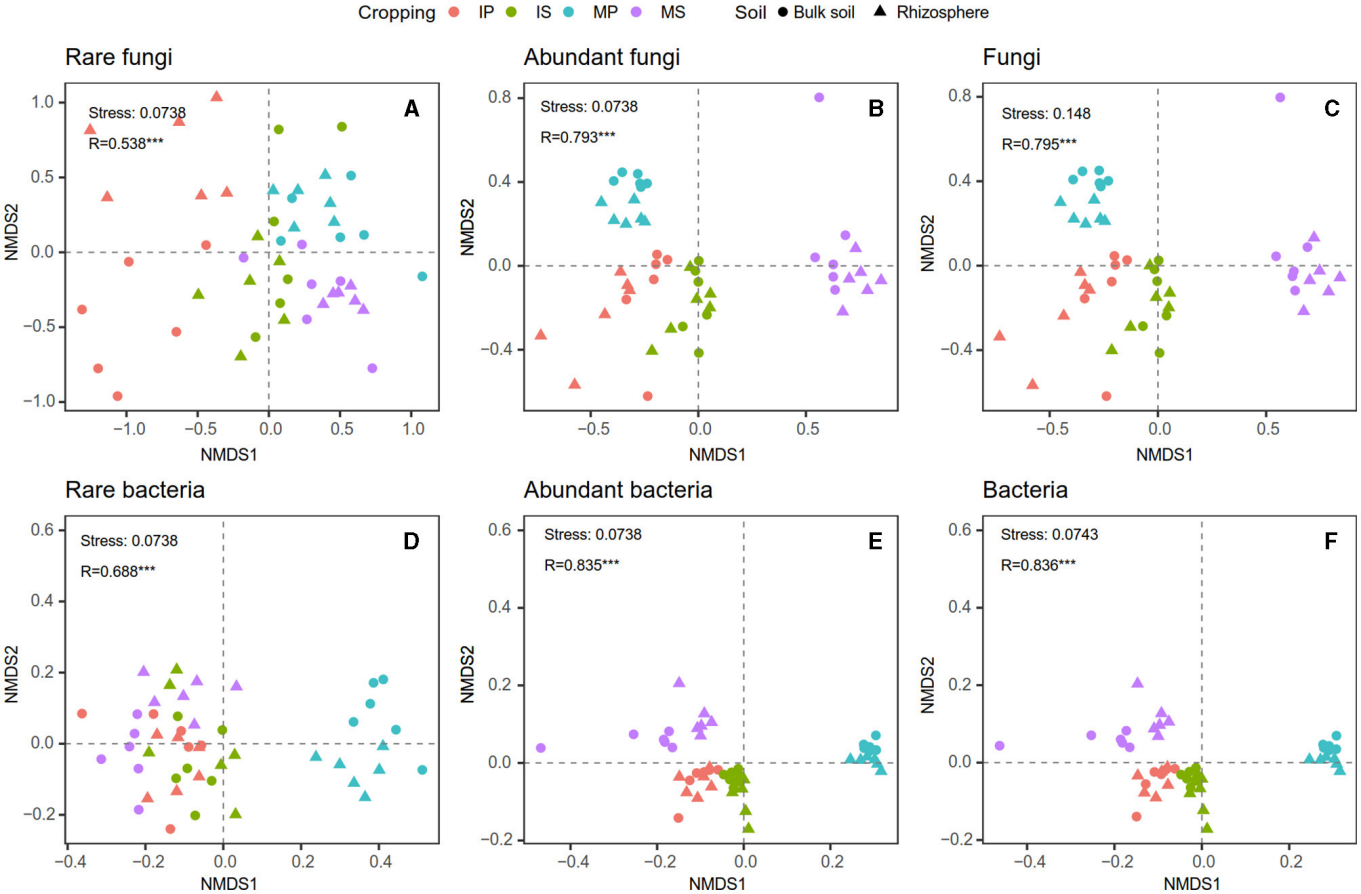
Intercropping effects on fungal **(A–C)** and bacterial **(D–F)** community beta diversity at phylum level based on non-metric multidimensional scaling (NMDS) analysis in the rhizosphere and bulk soil. MS, monoculture sugarcane; IS, intercropping sugarcane; MP, monoculture peanut; IP, intercropping peanut.

Compared with monoculture systems, intercropping increased MBC and MBC:MBN in the rhizosphere and bulk soil but did not alter MBN, MBP, MBC:MBP, and MBN:MBP (Supplementary Table S2). Intercropping increased abundant and total bacterial Shannon diversity in both rhizosphere and bulk soil (Figure 2). The Shannon diversity of abundant and total fungal taxa was higher in sugarcane than in peanut systems (Figure 2). The NMDS analysis showed that intercropping and rhizosphere affected rare and abundant taxa beta diversity at the phylum level, and the intercropping effects were stronger than rhizosphere effects (Figure 3). Across all soils, fungi were dominated by *Ascomycota* (>75%), *Basidiomycota, Mortierellomycota*, and unclassified phyla (Supplementary Figure S1A). Bacteria were dominated by *Actinobacteriota, Proteobacteria, Chloroflexi*, and *Acidobacteriota*, accounting for ~75% in total (Supplementary Figure S1B). Intercropping significantly altered the relative abundance of abundant taxa, including *Planctomycetota* and *Gemmatimonadota* in bulk soil and *Ascomycota* and *Actinobacteriota* in rhizosphere soil (Supplementary Figure S2). As for rare taxa, intercropping significantly affected the relative abundance of *Rozellomycota, Bdellovibrionota*, and *Armatimonadota* in bulk soil, and the relative abundance of *Methylomirabilota* and *Armatimonadota* in rhizosphere soil (Supplementary Figure S3). In addition, only the beta diversity of bacterial composition differed between rhizosphere and bulk soil (*P* < 0.05, Supplementary Table S3).

### 3.3 Intercropping effects on soil enzyme activity and stoichiometry in the rhizosphere and bulk soils

**Figure 4:**
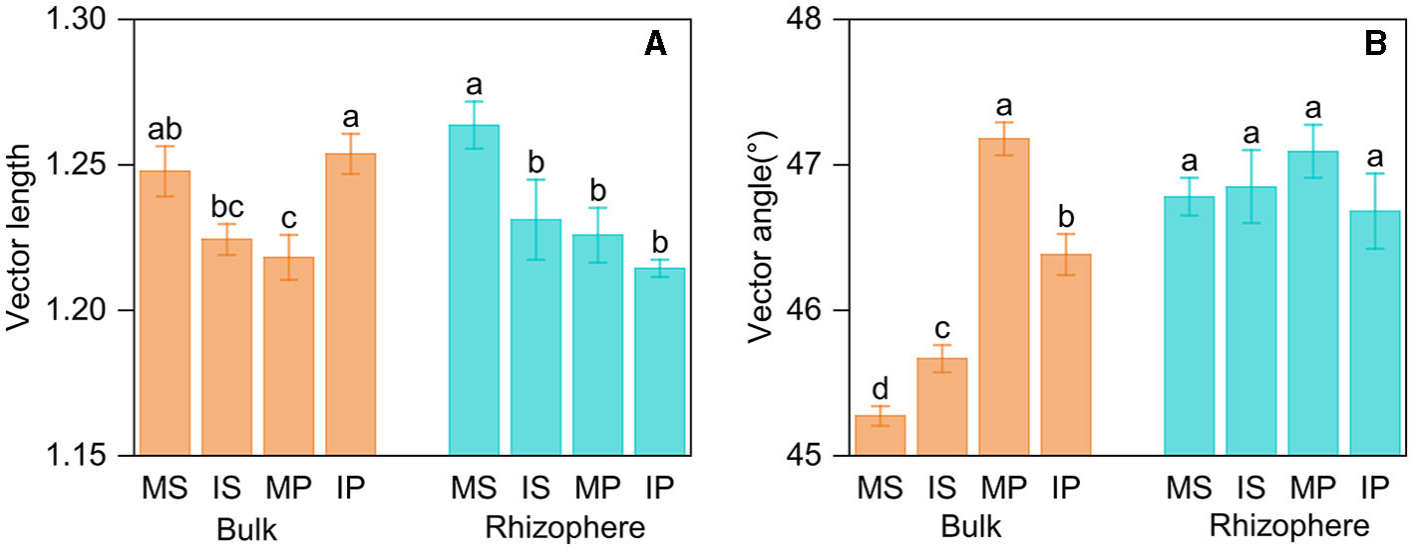
Vector length **(A)** and vector angle **(B)** based on soil enzyme stoichiometry in intercropping and monoculture systems. MS, monoculture sugarcane; IS, intercropping sugarcane; MP, monoculture peanut; IP, intercropping peanut. The lowercase and uppercase letters indicate significant intercropping effects in the rhizosphere and bulk soil by *LSD* test at *P* < 0.05, respectively.

Intercropping affected AP activity in bulk soil, but its effect differed in crop types. Intercropping decreased LAP activity in both rhizosphere and bulk soil, whereas decreased BG activity solely in rhizosphere soil (Supplementary Table S3). The ln (BG): ln (NAG+LAP) was more than 1:1, indicating microbes were limited by C relative to N. Based on the vector analysis of enzyme stoichiometry, intercropping decreased vector length in both rhizosphere and bulk soil (Figure 4A), and the vector angle was more than 45°, indicating microbes were limited by P relative to N (Figure 4B). Intercropping primarily impacted enzyme stoichiometry, vector length, and angle in bulk soil but not in rhizosphere soil (Supplementary Tables S3, S4, Figure 4). The enzyme activity, stoichiometry, and vector angle were also different between the rhizosphere and bulk soil, with higher BG, AP, ln (NAG+LAP): ln AP, and vector angle, as well as lower LAP and ln BG:ln AP in rhizosphere than those in bulk soil (Supplementary Tables S3, S4). Furthermore, crop type exerted an influence on vector length and angle, with higher vector length in sugarcane rhizosphere soil and higher vector angle in peanut bulk soil, respectively (Supplementary Table S4).

### 3.4 Relationship among soil properties, microbial community, and enzymes in the rhizosphere and bulk soils

**Figure 5:**
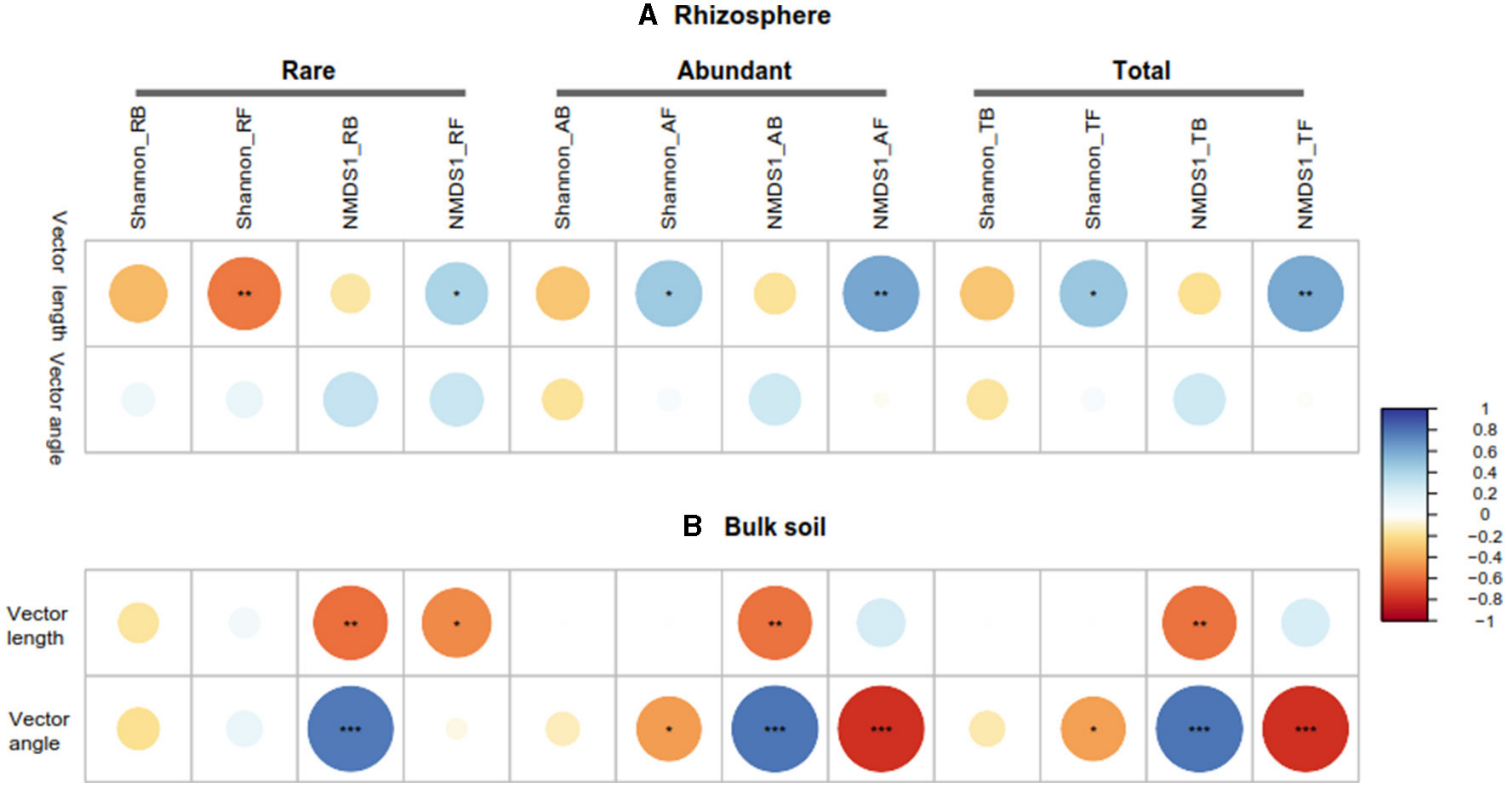
Pearson correlations between microbial community (rare, abundant, and total), vector length, and vector angle in rhizosphere **(A)** and bulk soil **(B)**. RB, RF, AB, and AF: the rare bacteria and fungi, abundant bacteria and fungi, respectively; NMDS1, the first component of the non-metric multidimensional scaling (NMDS) analysis of microbial community by bray-distance. ^*^P < 0.05; ^**^P < 0.01; ^***^P < 0.001.

**Figure 6:**
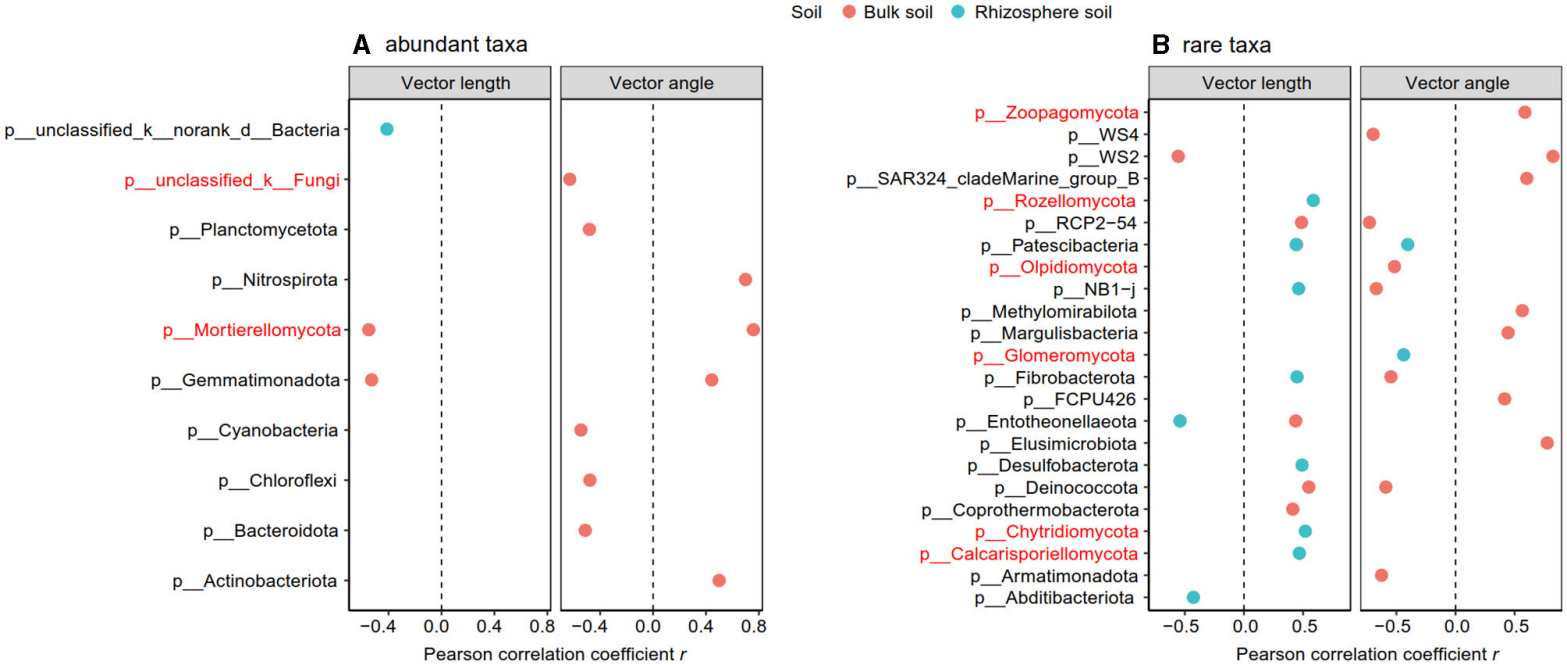
Significant Pearson correlation between abundant **(A)** and rare taxa **(B)** with vector length and angle in the rhizosphere and bulk soil. The taxa in red indicated the fungal group.

**Figure 7:**
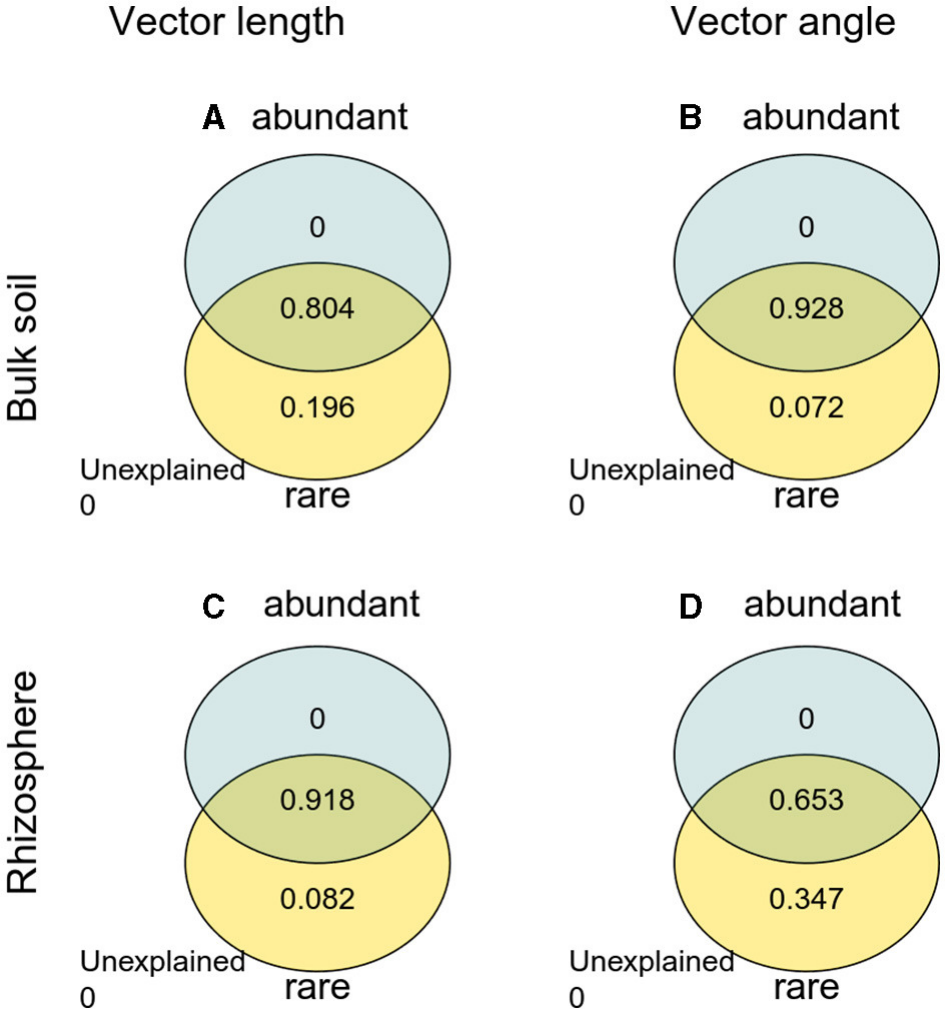
Variation partitioning analysis (VPA) of abundant and rare taxa with vector length and angle. **(A)** Vector length in bulk soil; **(B)** vector angle in bulk soil; **(C)** vector length in rhizosphere soil; **(D)** vector angle in rhizosphere soil.

**Figure 8:**
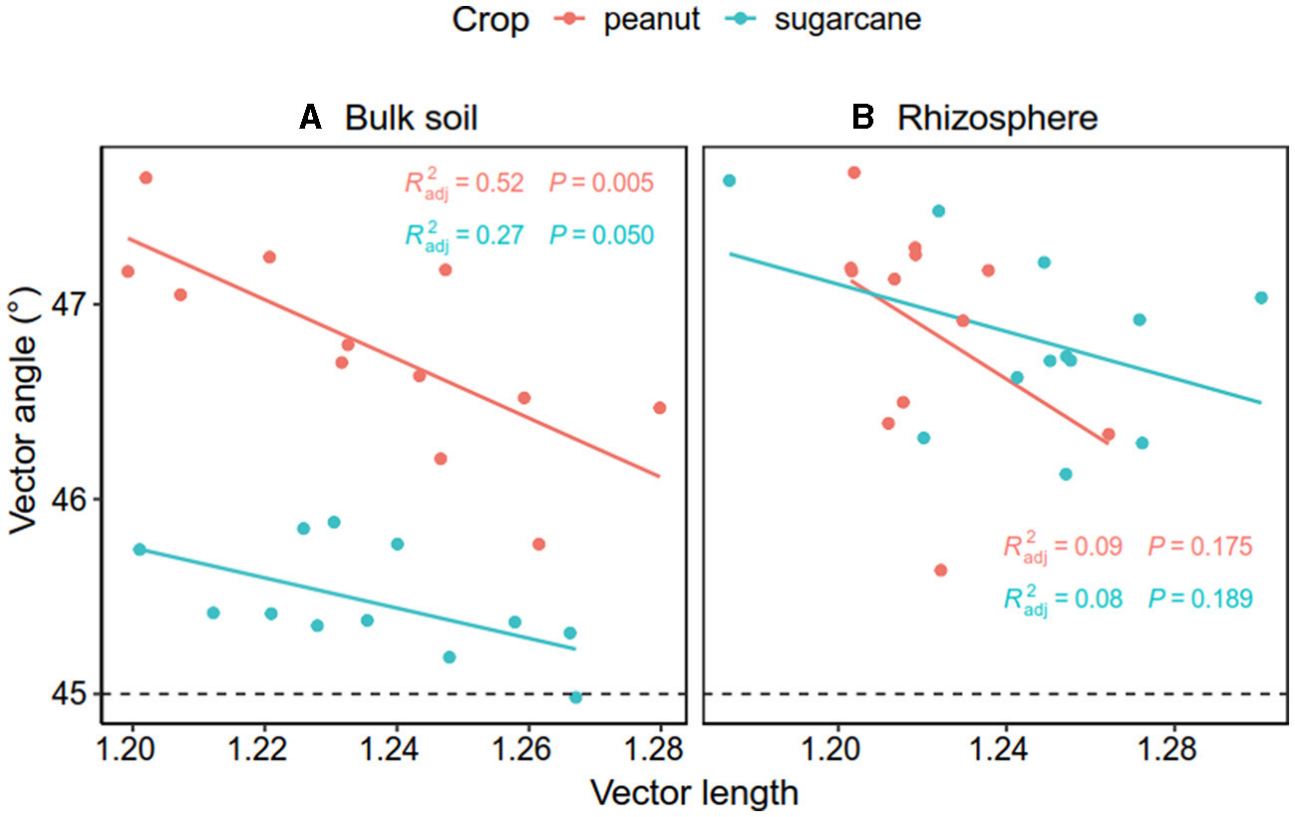
Relationship between vector length and angle in bulk soil **(A)** and the rhizosphere soil **(B)**. The red and green dots and lines indicate peanut and sugarcane soils, respectively.

The Pearson correlation showed that microbial communities of rare and abundant taxa and vector angle were significantly related to soil C:N and N:P ratio in bulk soil alone (Supplementary Figure S6). In rhizosphere soil, vector length and angle were primarily related to rare taxa, fungal Shannon, and beta diversity (Figures 5, 6). Vector angle was solely negatively related to *Patescibacteria* and *Glomeromycota* (Figure 6). In bulk soil, vector length was primarily and negatively related to bacterial diversity (Figure 5A) and was significantly related to abundant taxa (*Mortierellomycota* and *Gemmatimonadota*) and rare taxa (Figure 6A). Vector angle was primarily related to fungal and bacterial beta diversity, abundant taxa (*Nitrospirota* and *Mortierellomycota*), and most of the rare taxa at the phylum level in bulk soil (Figures 5, 6). Furthermore, the VPA analysis showed that the individual effects of rare taxa explained 7–35% of the variations, and the interactions of rare and abundant taxa explained 65–93% of variations in vector length and angle in both rhizosphere and bulk soil (Figure 7). The vector length had a negative correlation with vector angle in bulk soil but not in rhizosphere soil, especially in the sugarcane system ($R_{\text{adj}}^{2}$ = 0.52, *P* = 0.005; Figure 8).

## 4 Discussion

### 4.1 Intercropping effects on microbial C and nutrient limitation between rhizosphere and bulk soil

Rhizo-deposition significantly affects microbial metabolisms due to high nutrient availability and microbial activity (Gong et al., 2019; Cui et al., 2020). In this study, we measured microbial C and nutrient metabolism limitation in the rhizosphere and bulk soil based on enzyme stoichiometry and vector analysis. In this study, microbes were limited by C and P in both bulk and rhizosphere soil because of ln(BG)/ln(NAG+LAP) >1 and vector angle >45°. In contrast, microbial N limitation was alleviated especially intercropping with peanut through its biological N_2_ fixation function, which supports the view that intercropping peanut helps to promote soil N availability. We also observed that microbial P limitation was stronger in rhizosphere soil than in bulk soil, suggesting a faster P cycle in rhizosphere soil, in agreement with a previous study (Cui et al., 2021). This was ascribed to high demands of microbes on soil available P in rhizosphere soil where microbes are *r*-strategist copiotrophs and fast-growing, which require more P investments for biomass synthesis (ribosomal RNA), and thus have higher enzyme production for P-acquisition (Ren et al., 2021). The rhizosphere effects are stronger and regulate the intercropping effects on microbial P limitation. Intercropping had significant effects on microbial P limitation in bulk soils but not rhizosphere soils. Bulk soil is generally limited by nutrient and microbial activity, and the soil nutrient status is regulated by rhizosphere nutrient release and migration. Intercropping might shift bulk soil from oligotrophic into eutrophic soils and regulate bulk soil microbial community through stronger rhizo-deposition effects compared to sole crops. This result can be supported by higher bulk soil nutrient status and microbial diversity when intercropped than sole crops.

Microbial C limitation was alleviated by intercropping peanuts in the rhizosphere and bulk soil, suggesting the dependence of microbial C metabolisms on intercropping. Compared to sole crops, intercropping peanuts can increase soil N availability through biological N_2_ fixation and promote crop and microbial growth, consequently, increasing soil labile-C inputs and decreasing microbial C limitation. In contrast, microbial C limitation was almost unchanged between rhizosphere and bulk soil. Previous studies have revealed that the rhizosphere has various effects on microbial C limitation with positive, negative, and no impacts relative to bulk soil (Cui et al., 2021; Bi et al., 2022; Kang et al., 2022). These different rhizosphere effects on microbial C limitation might be attributed to the interactions of soil and microbial properties.

Crop type exerted an important influence on microbial C and P limitation (Supplementary Table S4). Microbial C limitation was alleviated in the peanut system than in the sugarcane system. In contrast, microbial P limitation was enhanced in the peanut system compared to the sugarcane system. This is because the peanut system has high N availability due to its biological N_2_-fixing function, thereby increasing microbial P demands. Meanwhile, the coupled soil C and N might explain the alleviated microbial C limitation in peanut systems where soil N is rich (Moorhead et al., 2016). We also found that the intercropping effects on microbial C and P limitation were crop-related in bulk soil. When intercropped, the N_2_ fixed by the peanut is gradually depleted by the surrounding soil, leading to a decrease in soil N availability within the peanut system. In contrast, sugarcane–peanut intercropping (IS treatment) increased soil N availability and promoted microbial growth and metabolism, particularly in bulk soil. Taken together, the differences in microbial C and P limitation across crops might be explained by the interspecific competition on soil N in the intercropping system (Feng et al., 2021).

### 4.2 Microbial community mediates the intercropping effects on microbial C and nutrient limitation between rhizosphere and bulk soil

The microbial community is the key to regulating ecosystem functions and services, such as soil nutrient and carbon cycles via altering microbial metabolisms (Delgado-Baquerizo et al., 2016; Maron et al., 2018). Supported by our hypothesis, both rare and abundant taxa diversity are vital to affecting microbial P and C limitation in bulk soil, which agreed with a previous study (Yang et al., 2021). Microbial P limitation was negatively related to fungal beta diversity in bulk soil, indicating that higher fungal community composition diversity helps to alleviate microbial P limitation. This may be attributed to the fact that complex microbial community structures can have diverse functionalities, such as decomposing and utilizing diverse soil substrates (Zhou Z. H. et al., 2020), which increase the decomposition of P compounds and satisfy microbial P requirements. In rhizosphere soil, however, microbial diversity did not affect microbial P limitation. One reason may be ascribed to the functional redundancy hypothesis in rhizosphere soil (Wertz et al., 2006). The rhizosphere, as a hotspot in soil, has high microbial diversity and multi-functionality in soil nutrient and C cycle processes (Ling et al., 2022). This hotspot leads to diverse microbial groups that share similar and even the same functions (functional redundancy), thereby weakening the contributions of microbial diversity to microbial metabolisms. Another possible reason was that microbial P limitation in rhizosphere soil was primarily regulated by rare taxa, including *Patescibacteria* and *Glomeromycota* at the phylum level. Phylum *Glomeromycota* (fungal community) generally increases soil P availability and P uptake, as well as improves the stress tolerance of their host plants (Liu et al., 2012; Procter et al., 2014).

The rare taxa, but not abundant ones, regulate microbial C and P limitation in both rhizosphere and bulk soil, indicating the importance of rare taxa in soil C and nutrient cycles (Han and Wang, 2023; Xu et al., 2024). This is because rare taxa have faster responses to stress and disturbance than abundant taxa, which can be a sensitive indicator of environmental changes (Xu et al., 2021a; Lin et al., 2022). In this study, compared to abundant taxa, rare taxa had a stronger correlation with microbial C and P limitation in all soils (Figure 5). Furthermore, microbial C limitation was primarily affected by rare bacterial taxa in bulk soil and by rare fungal taxa (*Rozellomycota, Chytridiomycota*, and *Calcarisporiellomycota*) in the rhizosphere (Figure 5). Fungi prefer plant-derived C compounds (e.g., root exudates), whereas bacteria prefer small molecular substrates (e.g., sugars) (de Boer et al., 2005; Paterson et al., 2008). Microbial substrate preferences lead to that fungi are prior to utilizing soil substrates in rhizosphere soil where root-derived C is rich (Sylvia et al., 2005). In bulk soil, however, bacterial beta diversity was strongly dependent on soil C:N and N:P ratios because of the nutrient imbalance (Supplementary Figure S6B). The imbalance of soil nutrients leads to changes in the bacterial community to utilize a wider variety of substrates through enzyme production (Liu et al., 2020). Thus, the substrate preferences explained the different responses of bacterial and fungal rare taxa to C acquisition in bulk and rhizosphere soil.

### 4.3 Relationships between microbial C and nutrient limitation in the rhizosphere and bulk soil

The breakdown of SOM often releases both C and P; therefore, soil C and P are generally coupled, and in turn, microbes produce C-related enzymes to meet C acquisition demands and alleviate P limitation as well. However, this coupling relationship varies with soil nutrient availability (Cui et al., 2020; Sun et al., 2023). For instance, a recent study observed that P/C–acquisition enzymes have a negative correlation in bulk soil but a positive correlation in rhizosphere soil where labile C is rich (Liu et al., 2021), which is partly consistent with our study. In this study, we observed that microbial P limitation (vector angle) had a negative correlation with microbial C limitation (vector length) solely in bulk soils. This implies that microbial P/C acquisition is coupled in bulk soil but not in rhizosphere soil. Root acidifying rhizosphere soil might be one possible reason for the no relationship between microbial C and P limitation. A meta-analysis indicates that compared with bulk soil, legumes induce a decrease of 0.44 unit in rhizosphere soil pH during biological N_2_ fixation (Liu et al., 2022), which could enhance soil P availability via the dissolution of Fe–P and Al–P complexes in acidic soils (Haynes, 1983; Liu et al., 1989) and reduces microbial P limitation. This is supported by the positive relationship between soil pH and microbial P limitation. Therefore, intercropping legumes alleviate microbial P limitation through enzyme production and soil acidification in rhizosphere soil (Liu et al., 2021), which explains the no correlation between microbial C and P limitation in rhizosphere soil.

## 5 Conclusion

In this study, microbes were limited by C and P in all soils. Microbial C limitation was alleviated by sugarcane–peanut intercropping, microbial P limitation was stronger in rhizosphere soil than in bulk soil, and was enhanced by sugarcane–peanut intercropping in bulk soil but not in rhizosphere soil due to root acidification. These results indicate that strong microbial P limitation promotes soil P decomposition and thus enhances P turnover in rhizosphere and intercropping systems. The intercropping effects on microbial C limitation were primarily regulated by microbial beta diversity and rare taxa in the rhizosphere and bulk soil. Microbial P limitation was mostly modified by the interactions of rare and abundant taxa in bulk soil and by rare taxa in rhizosphere soil. This study emphasizes the positive effects of intercropping on soil P cycles, and the importance of microbial community, in particular, rare taxa, in microbial nutrient limitation. In conclusion, intercropping peanuts facilitates soil nutrient cycles through accelerating microbial metabolisms (e.g., rare taxa), especially in P-limited soils.

## Data availability statement

The datasets presented in this study can be found in online repositories. The names of the repository/repositories and accession number(s) can be found here: https://www.ncbi.nlm.nih.gov/, PRJNA1051854.

## Author contributions

YF: Formal analysis, Investigation, Resources, Visualization, Writing—review & editing. XT: Methodology, Supervision, Writing—review & editing. TS: Conceptualization, Formal analysis, Funding acquisition, Investigation, Methodology, Resources, Supervision, Visualization, Writing—original draft, Writing—review & editing. LL: Investigation, Writing—original draft, Writing—review & editing. LW: Writing—original draft, Writing—review & editing. TZ: Writing—original draft, Writing—review & editing. YG: Writing—original draft, Writing—review & editing. YL: Writing—original draft, Writing—review & editing. HW: Writing—original draft. JX: Writing—original draft. RT: Supervision, Writing—original draft, Writing—review & editing.
