# Supplementary material for: Rare taxa mediate microbial carbon and nutrient limitation in the rhizosphere and bulk soil under sugarcane–peanut intercropping systems
Source: Front Microbiol. 2024 May 30;15:1403338. doi: 10.3389/fmicb.2024.1403338 (PMC11169858; doi:10.3389/fmicb.2024.1403338)
Supplement: Supplementary file 1 [file Data_Sheet_1.pdf]

# Rare taxa mediate microbial carbon and nutrient limitation in the rhizosphere and bulk soil under sugarcane-peanut intercropping systems

Yue Fu[a, b#], Xiumei Tang[c#], Litao Lin[d], Tingting Sun[a, b,*], Lixue Wu[a,b], Tian Zhang[a, b], Yifei Gong[a], Yuting Li [a],Haining Wu[c], Jun Xiong[c], Zhigang Huang[a,b,*], Ronghua Tang[c,*]

[a] *College of Agronomy, Guangxi University, Nanning, Guangxi, PR China*

[b] *Key Laboratory of Agro-Environment and Agro-Product Safety, Guangxi University, 530005, Nanning, China*

[c] *Guangxi Academy of Agricultural Sciences, Cash Crops Research Institute, Nanning, Guangxi, China*

[d] *Center for Ecological Civilization Research, Chinese Research Academy of Environmental Sciences, Beijing 100012, China*

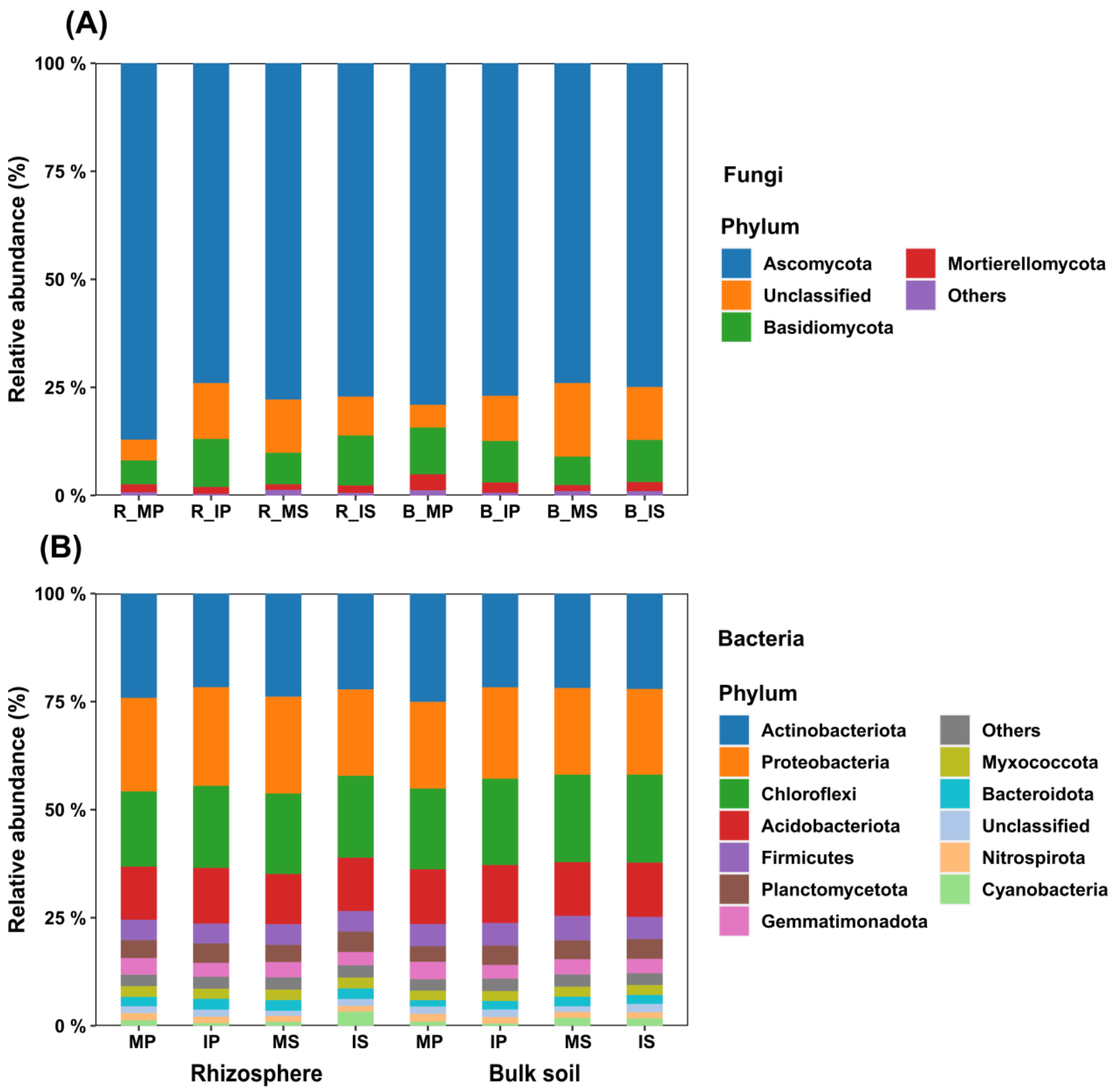


**Fig. S1** The relative abundance of fungal and bacterial community at the phylum level in different cropping systems in rhizosphere and bulk soil. MS: monoculture sugarcane; MP: monoculture peanut; IS: sugarcane soil in intercropping system; IP: peanut soil in intercropping system.

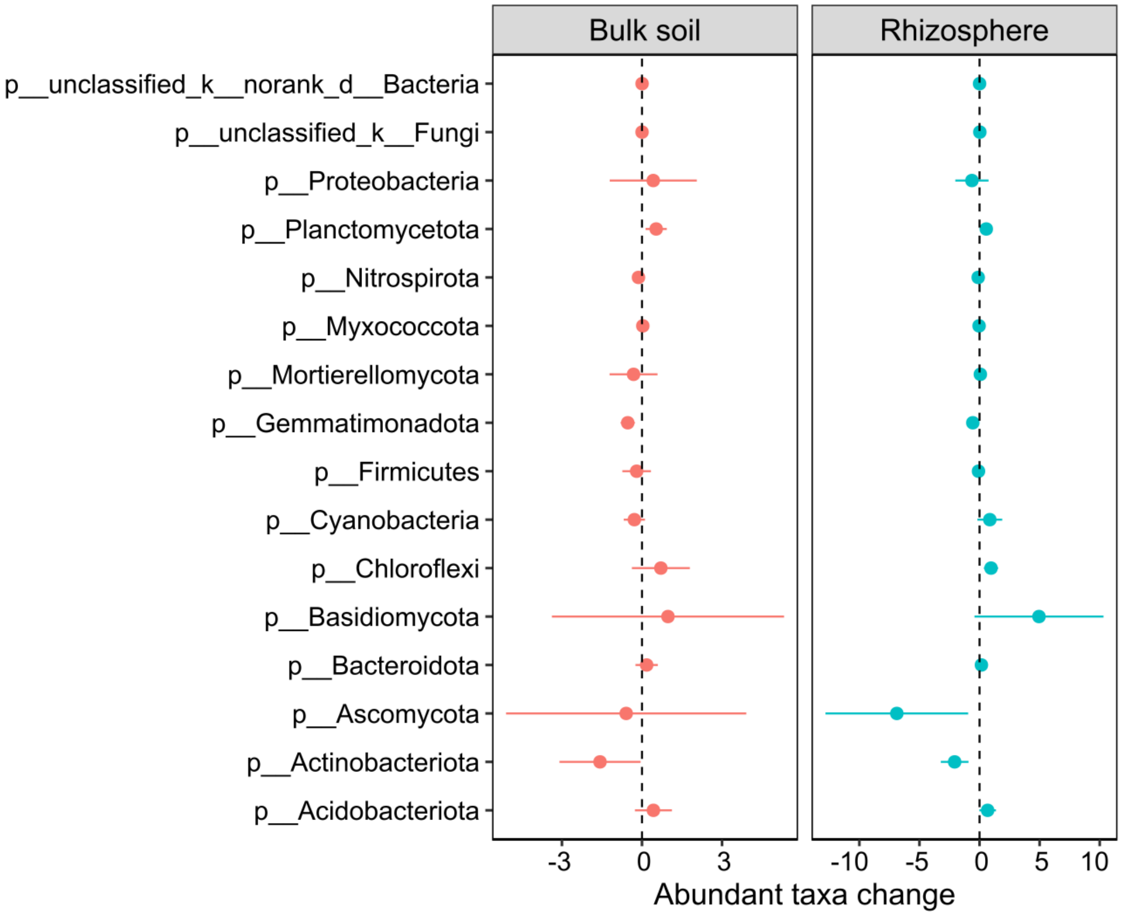


**Fig. S2** The absolute changes of abundant taxa at phylum level between intercropping and monoculture in rhizosphere and bulk soil. The error bar represents 95% confident intervals (CI). The CI cross zero indicates intercropping does not significantly affect taxa.

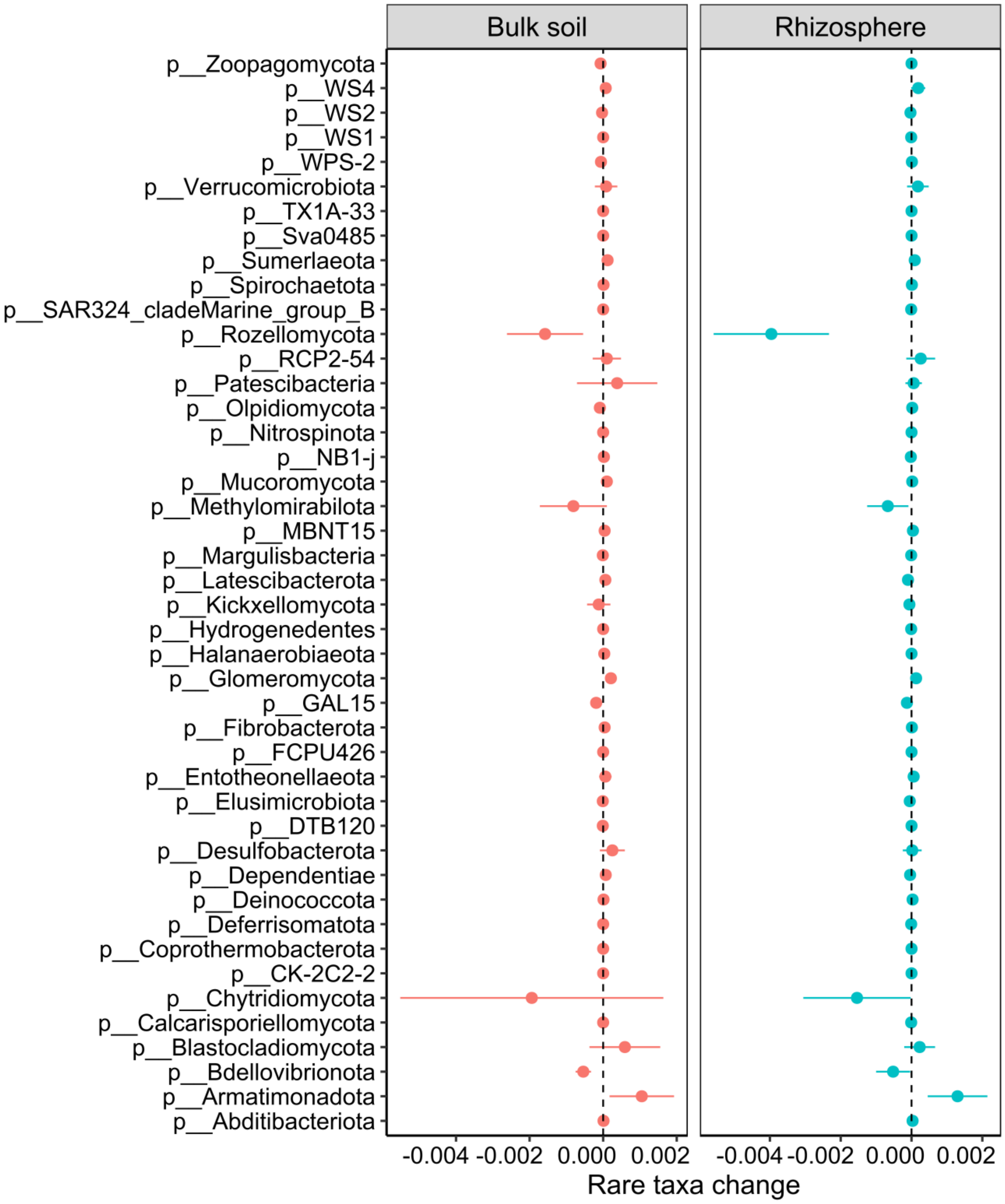


**Fig. S3** The absolute changes of rare taxa at phylum level between intercropping and monoculture in rhizosphere and bulk soil. The error bar represents 95% confident intervals (CI). The CI cross zero indicates intercropping does not significantly affect microbial taxa.

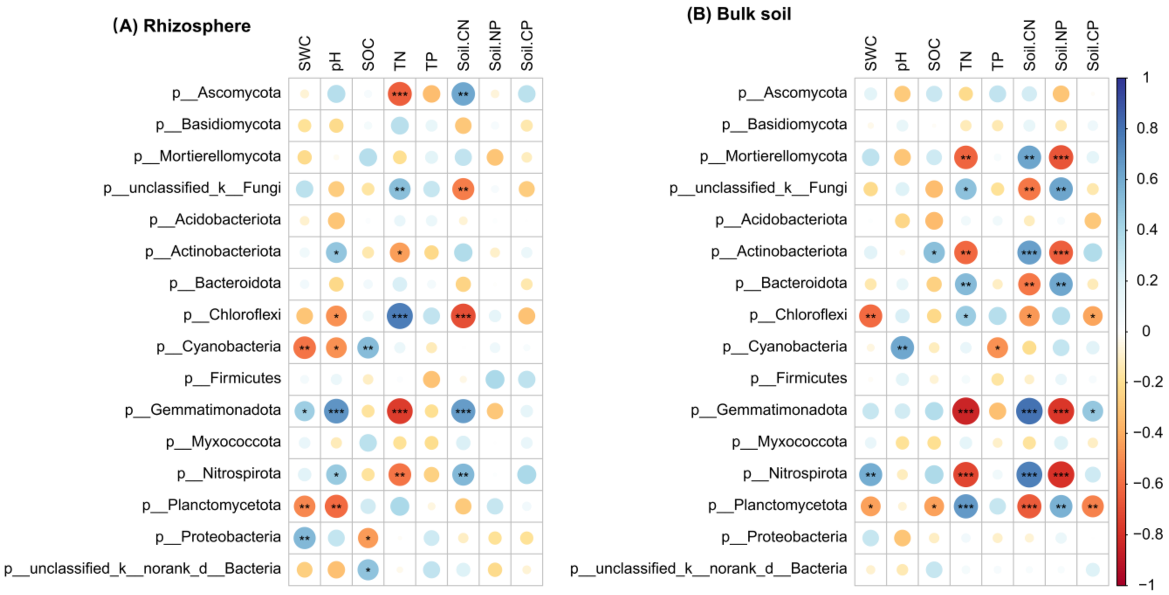


**Fig. S4** Pearson correlation of abundant taxa at phylum level with soil properties in rhizosphere and bulk soil. SWC: soil water content; SOC: soil organic carbon; TN: total nitrogen; TP: total phosphorus; Soil. CN: the ratio of soil organic carbon to nitrogen; Soil.NP: the ratio of soil nitrogen to phosphorus; Soil.CP: the ratio of soil organic carbon to phosphorus.

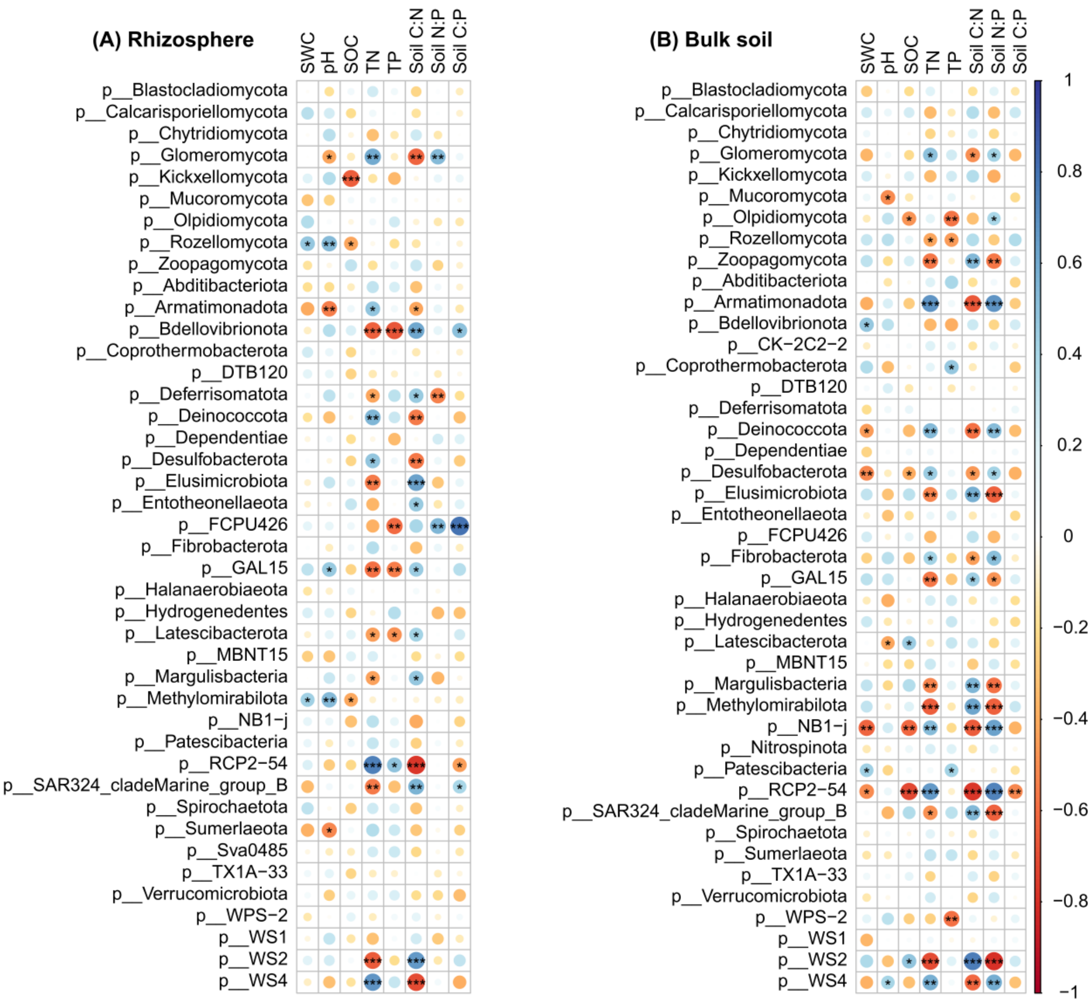


**Fig. S5** Pearson correlation of rare taxa at phylum level with soil properties in rhizosphere and bulk soil. SWC: soil water content; SOC: soil organic carbon; TN: total nitrogen; TP: total phosphorus; Soil. CN: the ratio of soil organic carbon to nitrogen; Soil.NP: the ratio of soil nitrogen to phosphorus; Soil.CP: the ratio of soil organic carbon to phosphorus.

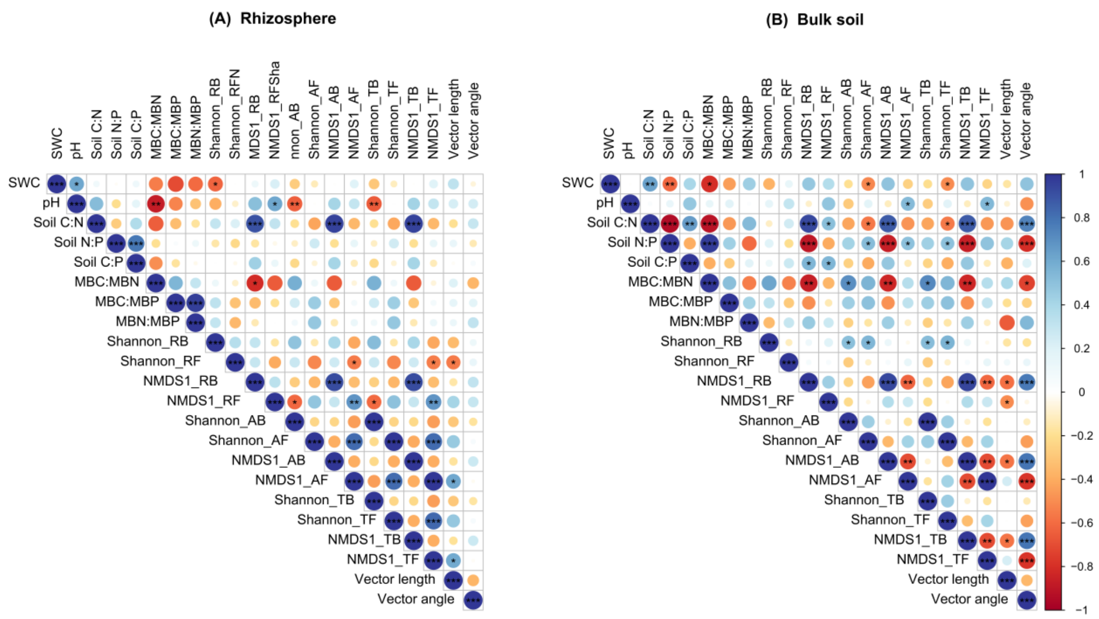


**Fig. S6** Pearson correlations among soil, microbial community, vector length and vector angle in rhizosphere (A) and bulk soil (B). SWC: soil water content,%; soil C:N, the ratio of soil organic carbon to total nitrogen; soil N:P, the ratio of soil nitrogen to phosphorus; soil C:P, the ratio of soil organic carbon to phosphorus; MBC:MBN, the ratio of microbial biomass carbon to nitrogen; MBC:MBP, the ratio of microbial biomass carbon to phosphorus; MBN:MBP, the ratio of microbial biomass nitrogen to phosphorus; RB, RF, AB, AF, TB, TF, the rare bacteria and fungi, the abundant bacteria and fungi, and the total bacteria and fungi; NMDS1, the first component of the non-metric multidimensional scaling (NMDS) analysis of microbial community.

**Table S1** The ANOVA analysis of soil physicochemical properties between crop type, soil and cropping systems.

| Soil | Cropping | pH | SWC (%) | SOC (g/kg) | TN (g/kg) | TP (g/kg) | Soil C:N | Soil N:P | Soil C:P |
|---|---|---|---|---|---|---|---|---|---|
| Rhizosphere | MS | 6.51±0.1a | 23.68±0.25a | 13.68±0.75c | 2.50±0.03b | 1.30±0.03b | 5.48±0.32bc | 1.93±0.05a | 10.60±0.69a |
| | IS | 4.07±0.18c | 20.77±0.25b | 16.79±0.37a | 2.77±0.05a | 1.29±0.01b | 6.09±0.22b | 2.15±0.03a | 13.05±0.38a |
| | MP | 6.76±0.06a | 22.96±0.32a | 15.44±0.46ab | 1.34±0.01c | 1.21±0.2b | 11.51±0.35a | 1.54±0.54a | 17.47±6.08a |
| | IP | 5.00±0.17b | 22.72±0.61a | 14.95±0.13bc | 2.85±0.01a | 1.62±0.01a | 5.24±0.05c | 1.76±0.01a | 9.22±0.08a |
| Bulk soil | MS | 6.87±0.02a | 21.70±0.20b | 13.91±0.52b | 2.52±0.02b | 1.30±0.05b | 5.52±0.19b | 1.95±0.06a | 10.79±0.53b |
| | IS | 6.69±0.19ab | 21.79±0.22b | 16.16±0.34a | 2.83±0.06a | 1.40±0.02ab | 5.72±0.13b | 2.02±0.06a | 11.52±0.28ab |
| | MP | 6.36±0.12b | 23.6±0.08a | 16.76±0.25a | 1.31±0.01c | 1.35±0.05b | 12.78±0.21a | 0.98±0.03b | 12.48±0.52a |
| | IP | 5.68±0.16c | 21.3±0.89b | 14.03±0.55b | 2.82±0.01a | 1.49±0.04a | 4.97±0.17c | 1.90±0.04a | 9.45±0.33c |
| Crop type | | ns | * | ns | ** | ns | ** | ** | ns |
| Soil | | ** | ns | ns | ns | ns | ns | ns | ns |
| Cropping | | ** | ** | ns | ** | ** | ** | * | ns |
| Crop type: Soil | | ** | ns | ns | ns | ns | * | ns | ns |
| Crop type: Cropping | | ns | ns | ** | ** | * | ** | ns | * |
| Soil: Cropping | | ** | ns | * | ns | ns | ** | ns | ns |
| Crop type: Soil: Cropping | | ** | ** | ns | ns | ns | ns | ns | ns |

Note: data is expressed as means ± standard errors (se, n=6). MS: sugarcane monoculture, IS: sugarcane soil in intercropping system s, MP: peanut monoculture, IP: peanut soil in intercropping systems. SWC: soil water content, SOC: soil organic carbon, TN: total nitrogen,

TP: total phosphorus. Soil: rhizosphere vs. bulk soil; Cropping: monoculture vs. intercropping; Crop type: sugarcane vs. peanut. Different lowercase letters indicate significant differences among cropping treatments by *LSD* test. Significance level: ns: not significant; *: $P < 0.05$; **: $P < 0.01$.

**Table S2** The ANOVA analysis of soil microbial biomass properties between crop type, soil and cropping systems

| Soil | Cropping | MBC (mg/kg) | MBN (mg/kg) | MBP (mg/kg) | MBC: MBN | MBN: MBP | MBC: MBP |
|---|---|---|---|---|---|---|---|
| Rhizosphere | MS | 122.45±4.35b | 20.97±1.59a | 31.14±5.44a | 5.89±0.39b | 0.70±0.10a | 4.17±0.68ab |
| | IS | 186.20±7.25a | 24.28±0.19a | 28.14±9.77a | 7.67±0.25a | 1.07±0.32a | 8.08±2.19a |
| | MP | 108.02±9.10b | 23.86±2.81a | 40.08±4.53a | 4.58±0.33c | 0.59±0.03a | 2.71±0.09b |
| | IP | 166.21±10.12a | 24.17±1.31a | 44.55±10.12a | 6.87±0.18a | 0.61±0.15a | 4.16±0.96ab |
| Bulk soil | MS | 133.09±14.87a | 19.07±1.70b | 25.91±5.87a | 6.95±0.16b | 0.83±0.22a | 5.83±1.55a |
| | IS | 163.9±9.28a | 20.45±1.01b | 29.31±2.82a | 8.01±0.20a | 0.71±0.08a | 5.67±0.49a |
| | MP | 128.1±13.17a | 33.1±5.12a | 31.98±4.38a | 3.93±0.25c | 1.03±0.02a | 4.05±0.24a |
| | IP | 163.39±13.88a | 22.19±1.11b | 29.71±1.4a | 7.34±0.25ab | 0.75±0.06a | 5.55±0.65a |
| Crop type | | ns | * | ns | ** | ns | * |
| Soil | | ns | ns | ns | ns | ns | ns |
| Cropping | | ** | ns | ns | ** | ns | * |
| Crop type: Soil | | ns | ns | ns | * | ns | ns |
| Crop type: Cropping | | ns | * | ns | ** | ns | ns |
| Soil: Cropping | | ns | ns | ns | ns | ns | ns |
| Crop type: Soil: Cropping | | ns | ns | ns | * | ns | ns |

Note: data are expressed as means ± standard errors (se, n=6). MS: sugarcane monoculture; IS: sugarcane intercropping; MP: peanut monoculture; IP: peanut intercropping. MBC: microbial biomass carbon; MBN: microbial biomass nitrogen; MBP: microbial biomass phosphorus; Soil: rhizosphere vs. bulk soil; Cropping: monoculture vs. intercropping; Crop type: sugarcane vs. peanut. The lowercase letters indicate significant

differences ($P < 0.05$) among rhizosphere and bulk treatments in different cropping systems by LSD. Significance level: ns: not significant; *: $P < 0.05$; **: $P < 0.01$.

**Table S3** Soil extracellular enzyme activities under different cropping patterns

| Soil | Cropping | BG (nmol/h/g) | NAG (nmol/h/g) | LAP (nmol/h/g) | AP (nmol/h/g) | In BG: In (NAG+LAP) | In (NAG+LAP): In AP | In BG: In AP |
|---|---|---|---|---|---|---|---|---|
| Rhizosphere | MS | 186.18±10.88a | 80.7±7.68a | 23.26±0.54b | 230.49±5.41a | 1.13±0.03a | 0.85±0.01a | 0.96±0.01a |
| | IS | 156.25±17.34a | 90.00±7.81a | 24.68±0.87b | 232.92±10.96a | 1.06±0.02ab | 0.87±0.01a | 0.92±0.02ab |
| | MP | 198.63±16.11a | 122.65±7.88a | 38.66±2.06b | 358.82±16.13a | 1.04±0.01b | 0.86±0.01a | 0.9±0.01b |
| | IP | 137.30±6.36b | 99.02±9.69a | 27.34±0.87c | 229.47±10.71b | 1.08±0.02b | 0.89±0.01a | 0.96±0.01b |
| Bulk soil | MS | 112.34±6.54b | 60.25±1.76b | 31.39±0.76a | 102.11±1.99c | 1.04±0.01ab | 0.98±0.01a | 1.02±0.01a |
| | IS | 111.64±4.06b | 72.69±6.09ab | 34.09±1.59a | 135.39±5.02b | 1.01±0.01b | 0.95±0.01b | 0.96±0.01b |
| | MP | 185.04±13.51a | 112.2±9.65a | 45.77±1.92a | 355.57±12.9a | 1.03±0.01b | 0.86±0.01d | 0.89±0.01c |
| | IP | 176.55±10.04a | 94.13±10.09a | 28.23±0.79c | 216.78±5.76b | 1.08±0.02a | 0.89±0.01c | 0.96±0.01b |
| Crop type | | ** | ** | ** | ** | ns | ** | ** |
| Soil | | ** | * | ** | ** | ns | ** | ** |
| Cropping | | ** | ns | ** | ** | ns | ns | ns |
| Crop type: Soil | | ** | ns | * | ** | ** | ** | ns |
| Crop type: Cropping | | ns | ** | ** | ** | ** | ns | ** |
| Soil: Cropping | | * | ns | ns | ns | * | ns | ns |
| Crop type: Soil: Cropping | | ns | ns | * | ns | ns | ns | * |

Note: data are expressed as means ± standard errors (se, n=6). MS: sugarcane monoculture; IS: sugarcane intercropping; MP: peanut monoculture; IP: peanut intercropping. Soil: rhizosphere vs. bulk soil; Cropping: monoculture vs. intercropping; Crop type: sugarcane vs. peanut. BG: β-1,4-glucosidase; NAG: β-1,4-N-acetylglucosaminidase; LAP: leucine aminopeptidase; AP: acid phosphatase. The lowercase letters indicate significant differences ($P < 0.05$) among rhizosphere and bulk treatments in different cropping patterns by LSD. Significance level: ns: not significant; *:

$P < 0.05$; **: $P < 0.01$.

**Table S4** The ANOVA analysis of microbial diversity, vector length and angle under different crop type, soil and cropping systems

| Variables | Crop type | Soil | Cropping | Crop type: soil | Crop type: cropping | Soil: cropping | Crop type: soil: cropping |
|---|---|---|---|---|---|---|---|
| Shannon_RB | 0.739 | 0.091 | 0.018 | 0.156 | 0.001 | 0.815 | 0.147 |
| Shannon_RF | 0.189 | 0.273 | 0.273 | 0.242 | 0.762 | 0.052 | 0.791 |
| Shannon_AB | 0.780 | 0.173 | <0.001 | 0.366 | 0.776 | 0.574 | 0.939 |
| Shannon_AF | <0.001 | 0.644 | 0.485 | 0.439 | 0.103 | 0.061 | 0.642 |
| Shannon_TB | 0.848 | 0.164 | <0.001 | 0.366 | 0.726 | 0.599 | 0.992 |
| Shannon_TF | <0.001 | 0.690 | 0.576 | 0.417 | 0.116 | 0.060 | 0.688 |
| NMDS1_RB | <0.001 | 0.189 | <0.001 | 0.037 | <0.001 | 0.723 | 0.008 |
| NMDS1_RF | <0.001 | 0.989 | <0.001 | 0.894 | <0.001 | 0.963 | 0.006 |
| NMDS1_AB | <0.001 | 0.008 | <0.001 | 0.004 | <0.001 | 0.045 | 0.045 |
| NMDS1_AF | <0.001 | 0.088 | <0.001 | 0.001 | <0.001 | <0.001 | 0.740 |
| NMDS1_TB | <0.001 | 0.007 | <0.001 | 0.004 | <0.001 | 0.049 | 0.039 |
| NMDS1_TF | <0.001 | 0.078 | <0.001 | 0.001 | <0.001 | 0.001 | 0.750 |
| Vector length | 0.026 | 0.706 | 0.188 | 0.028 | 0.002 | 0.023 | 0.119 |
| Vector angle | <0.001 | <0.001 | 0.124 | <0.001 | 0.001 | 0.899 | 0.143 |

Note: Soil: rhizosphere vs. bulk soil; Cropping: monoculture vs. intercropping; Crop type: sugarcane vs. peanut. Shannon_RB, Shannon_RF Shannon_AB, Shannon_AF, Shannon_TB, and Shannon_TF indicate the Shannon diversity of rare bacteria and fungi, abundant bacterial and fungi, and total bacteria and fungi, respectively. NMDS1, the first component of the non-metric multidimensional scaling (NMDS) analysis of microbial community by bray-distance.
